# Improved Anti-Tumour Adaptive Immunity Can Overcome the Melanoma Immunosuppressive Tumour Microenvironment

**DOI:** 10.3390/cancers11111694

**Published:** 2019-10-31

**Authors:** Nana Dang, Mark Waer, Ben Sprangers, Yuan Lin

**Affiliations:** 1 Laboratory of Molecular Immunology, Department of Microbiology, Immunology and Transplantation, Rega Institute for Medical Research, KU Leuven, 3000 Leuven, Belgium; nana.dang@kuleuven.be (N.D.); mark.waer@kuleuven.be (M.W.); 2 Department of Nephrology, University Hospitals Leuven, 3000 Leuven, Belgium

**Keywords:** melanoma, metastasis, immunogenicity, allogenic immunity

## Abstract

Clinical benefits obtained from checkpoint blockade regimens demonstrate the importance of overcoming the immunosuppressive tumour microenvironment (TME) in cancer immunotherapy. Intravenous (i.v.) injection of B16 melanoma cells (H-2K^b^) leads to lethal disseminated pulmonary metastasis in Balb/c recipients (H-2K^d^). This lack of immune control is related to low major histocompatibility complex (MHC) expression on B16 cells which is associated with delayed and decreased anti-tumour adaptive immune responses (e.g., alloantibody formation) as: (i) other tumour types with normal H-2K^b^ expression are rejected with concomitant antibody production; (ii) preincubation of B16 with IFN-gamma to upregulate H-2K^b^ expression resulted in improved antibody production and anti-tumour activity. The delayed/decreased anti-tumour adaptive immune responses induced by B16 inoculation is not able to interrupt progression of primary metastases, while it is able to effectively eliminate secondary inoculated subcutaneously (s.c.) B16 cells from progression. This is due to the presence of an immunosuppressive TME within the primary metastases characterized by increased regulatory T cells (Tregs) and an increased T helper cells (Th) 2/1 profile. These tumour-induced immunosuppressive T cell populations are counteracted by improved adaptive immunity via active and passive immunization, resulting in effective elimination of the TME, destruction of the metastatic tumour and a reversal of Th2/1 profile in a time-sensitive manner. Thus, we here demonstrate that the TME is not irreversible and adaptive immunity is able to eradicate established solid tumour and its immunosuppressive TME. This study will help design treatments to overcome the immunosuppressive effect of the TME and improve efficacy of cancer immunotherapy.

## 1. Introduction

Active immunosurveillance and elimination of malignant cells are central in the prevention of cancer development by the immune system. Tumour cells evade anti-tumour immunity via a process known as immune-editing [1,2,3] and the growth of solid tumours is often associated with the development of an immunosuppressive tumour microenvironment (TME) [4,5]. The development of metastases is consistently associated with a poor prognosis and remain the major cause of morbidity and mortality related to cancer in the clinic [5,6]. The TME consists of complex cellular and soluble components including not only malignant cells but also tumour infiltrating immune cells such as immune regulatory cells (e.g., Tregs) and tumour cell surrounding stromal cells [4]. The crosstalk of these cells with tumour cells influences tumour initiation, progression and metastasis [4]. Administration of immune checkpoint blockers such as monoclonal antibodies to PD-1/PD-L1 or CTLA-4, have yielded significant clinical benefits in a subset of melanoma patients, eventually leading to eliminate all tumour cells through specific tumour immunity [1,2,3]. Specifically, the efficacy of checkpoint blockade therapies depends mainly on the interaction of CD8 cytotoxic T lymphocytes (CTLs) and tumour antigens presented by major histocompatibility complex class I (MHC-I) molecules [7,8,9,10].

MHC-I molecules are necessary to present endogenous antigenic peptides to CTLs to allow for recognition and lysis of target cells [10]. Recently, an increasing number of tumour-associated antigens (TAAs) have been identified [11,12] and using them as immunotherapeutic targets has yielded promising preliminary results in experimental animal models and clinical trials [3,11,12,13]. However, a decrease or loss of MHC-I expression in certain human tumours (e.g., melanoma and small-cell lung cancer) has been shown to impede CTL-mediated anti-tumour immunity [10,11,12,14]. Adaptive immune responses are able to effectively eliminate non-self MHC expressing cells such as allogenic cells or organ transplants [10]. Compared to the immune response to autologous tumour, allogenic immune responses are not only powerful but also easily manipulated [10,15,16,17,18]. It becomes more and more clear that anti-tumour immunity depends on both innate and adaptive immune elements [10,11,12]. To focus on the latter, here, we used an allogenic tumour model where immune reactivity directed at alloreactive antigens, which is similar to adaptive anti-tumour immunity predominantly directed at tumour associated peptides presented on tumour MHC-I antigens. 

## 2. Materials and Methods

### 2.1. Cell Lines and Mice

The B16 melanoma cell line and T cell lymphoma cell line EL-4 were obtained from ATCC (Rockville, MD, USA). The colon carcinoma cell line MC38 was kindly provided by Prof. Max Mazzone (VIB-KU Leuven Centre for Cancer Biology, Leuven, Belgium). Tumour cells were cultured in DMEM (Gibco) supplemented with 10% heat-inactivated foetal calf serum (FCS), 2 mM L-glutamine, 100 U/mL penicillin and 100 g/mL streptomycin (Gibco) under standard conditions. Before the harvesting of B16 cells, a trypsin–EDTA solution was added to the culture flask for 5 min at 37 °C in a 5% CO_2_ to detach the tumour cells. Prior to their use, cells were tested for the presence of endotoxins with the LAL Chromogenic Endotoxin Quantitation Kit (Pierce) and for mycoplasma with PlasmoTest (InvivoGen) [5].

Male BALB/cJ mice and C57BL/6 mice were purchased from Janvier Labs (France) and 8-week-old mice were used for the experiments. All experiments were performed in accordance with the ethical committee approval (P159-2017) obtained from the Animal Ethics Committee of KU Leuven.

### 2.2. B16 Transplantable Tumour Mouse Models

Variable amounts of viable tumour cells at a concentration of 5 × 10^4^ in 100 µL PBS were injected i.v. via the tail vein of Balb/c mice. Pulmonary tumour cell growth was inspected by autopsy at specified time points following tumour inoculation. In order to evaluate the immune response elicited by i.v. tumour inoculation, mice were challenged subcutaneously (s.c.) with 1 million B16 cells in the right flank 14 days after the primary i.v. tumour injection. The subcutaneous outgrowth of melanoma tumours was evaluated by direct measurement of length, width and depth of the black subcutaneous protuberance with a calliper for up to a maximum of 40 days. The tumour volume was calculated by the formula (tumour volume = length (mm) × width^2^ (mm)/2). When the tumour volume exceeded 2000 mm^3^, mice were euthanized.

### 2.3. Active and Passive Immunization 

To overcome the poor immune response underlying the progression of B16 melanoma in allogenic recipients, Balb/c mice were immunized with C57BL/6 spleen derived-peripheral blood mononuclear cells (PBMC). 5 × 10^6^ PBMC in 100 µL PBS were injected intraperitonially (i.p.) at different time points as indicated.

Donor-specific CTLs (20 × 10^6^ in 200 µL PBS) generated from bulk MLR or donor-specific serum (200 µL) from immunized Balb/c mice or a combination of both were injected i.v. in the tail vein of Balb/c mice every other day starting from day 0 of i.v. tumour injection with a total 3 doses.

### 2.4. Flow Cytometry

Expression of MHC molecule on B16, EL-4 and MC38 cells was quantified by direct immunostaining and flow cytometry. Evaluation of upregulation of MHC antigens on B16 cells was performed by incubating cells with IFN-γ (20 µg/mL) overnight. To evaluate the expression of MHC molecules on metastatic melanoma in vivo, both lungs were removed 2 weeks after i.v. tumour injection, mechanically homogenized and filtered through a cell strainer (70 µM) to obtain a single cell suspension. B16 cells were stained with fluorescein isothiocyanate (FITC)-conjugated anti-H-2K^b^ (C57BL/6) and contaminating Balb/c cells were excluded based on the expression of H-2K^d^ (Balb/c) detected by staining with phycoerythrin (PE)-conjugated anti-H-2K^d^. 

Direct immunofluorescence using the following antibodies CD3, CD4, H-2K^b^ and H-2K^d^ (eBioscience) was performed as previously described [5]. Intracellular staining for Foxp3 (FJK-16s) was performed using the Foxp3 Transcription Factor Staining Buffer Set according the manufacturer’s instructions (eBioscience). Samples were acquired with a FACSCalibur flow cytometer (BD Biosciences) and analysed with Cell Quest (version 10.1; Tree Star).

Indirect immunofluorescence was used to quantify serum anti-H-2K^b^ antibody IgG levels. In brief, serum was incubated with target cells expressing H-2K^b^ antigens, followed by staining with goat anti-mouse IgG antibodies coupled to (FITC) (Cappel Laboratories). The samples were acquired using a FACSCalibur flow cytometer (BD Biosciences).

To evaluate the production of Th1/Th2 cytokines, mice bearing metastatic melanoma with or without allogenic PBMC immunization were stimulated in vivo with anti-CD3 antibodies (1 μg in 200 μL normal saline per mouse) by i.p. injection. Three hours later, serum IL-2 and IL-10 were quantified using flow cytometry-microbeads (Microbeads Bioscience, Boston, MA, USA) according to the manufacturer’s instructions.

### 2.5. In Vitro Adaptive Immunity against Tumour Cells

CTLs were generated by a standard one-way bulk MLR. In brief, mitomycin C-inactivated C57BL/6 splenocytes were used as stimulator cells and Balb/c splenocytes as responder cells at a ratio of 1:1 in 25 mm^2^ culture flasks. Following 3 days of cultures, the cells were harvested. CTLs were enriched by gradient centrifugation using Lymphoprep^TM^ (STEMCELL Technologies). Lymphokine activated killer (LAK) cells were induced by incubation of Balb/c splenocytes with recombinant mouse IL-2 (10 µg/mL) for 2 days. Target B16 cells were stimulated to up-regulate MHC class I molecules by pre-treatment with mouse recombinant IFN-γ (rIFN-γ; 20 µg/mL) overnight and used in all cytotoxicity assays. CTL-mediated cytotoxicity was evaluated by incubating CTLs with target B16 cells at a ratio of 20:1 for 4 h. LAK cell-mediated cytotoxicity was evaluated using the ADCC assay by incubating LAK cells with anti-H-2K^b^ serum coated target B16 cells at a ratio of 20:1 for 4 h incubation. The ratio of effector: target cells of 20:1 was selected based on pre-titration.

Humoral cytotoxicity was evaluated using the CDC assay. Serum of immunized mice (1:20 dilution in PBS) was incubated with target B16 cells for 30 minutes at 4 °C. Subsequently, these cells were washed twice with ice cold PBS. The cells were incubated in the presence of rabbit serum as a source of complement (1:20 dilution). 

Necrotic target cells as a consequence of cytotoxicity were stained by propidium Iodide (PI) penetration and quantified by flow cytometry.

### 2.6. Statistics 

Data are expressed as means ± SD. A two-tailed Student’s *t* test was used when only two variables were present in the analysis. Two-way ANOVA with multiple comparisons was used to compare tumour growth over time. Survival curves were estimated using the Kaplan-Meier method. A *p-value* of <0.05 was considered statistically significant. In the figures throughout the manuscript, a single asterisk (*) denotes *p* < 0.05, two asterisks (**) for *p* < 0.01, three asterisks (***) for *p* < 0.001 and four asterisks (****) for *p* < 0.0001. Analyses were performed using GraphPad Prism software.

## 3. Results 

### 3.1. Disseminated Pulmonary Melanoma Metastasis in Allogenic Balb/c Mice 

**Figure 1:**
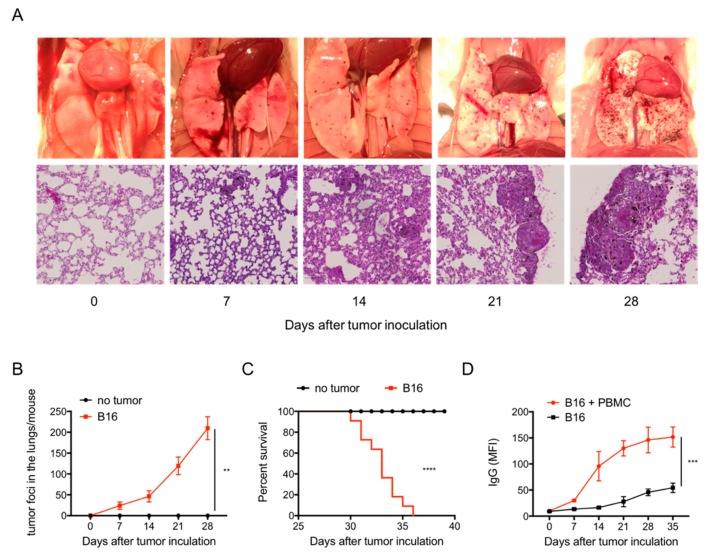
Disseminated pulmonary melanoma metastasis in allogenic Balb/c mice. (**A**) Clinical image showed that metastatic tumour foci started to occur 7 days post i.v. inoculation and increased in a time dependent manner (upper panel); Hematoxylin & Eosin (H&E) staining showed morphology of tumour foci and associated inflammation including the immune cell infiltration, alveolar wall thickness, interstitial oedema and fibrosis (lower panel). (**B**) Quantification of tumour foci in the lungs of Balb/c mice. B16 cell i.v. injection (■) vs. no tumor injection (●) (*p* < 0.01). (**C**) Animal survival after B16 cell i.v. injection. B16 cell i.v. injection (■) vs. no tumor injection (●) (*p* < 0.001). (**D**) anti-donor allo-antibody IgG in the blood. B16 i.v. plus peripheral blood mononuclear cells (PBMC) i.p. injection (●) vs. B16 i.v. injection (■) (*p* < 0.0001). *n* = 10 mice in each group.

Following i.v. injection, B16 melanoma cells are readily trapped in the lungs of allogenic Balb/c mice resulting in a progressive increase in number and volume of pulmonary tumour foci indicating this is a suitable model to study the evolution of experimental metastasis. Tumour foci appeared from 7 days post-i.v. tumour inoculation onwards and progressively increased with a mean ± SD of foci ranging from 19.2 ± 9.1 at day 7 up to 201.6 ± 29.2 at day 28 (Figure 1A,B). Histopathologic evaluation showed a time dependent increase in tumour foci and inflammation (immune cell infiltration, alveolar wall thickness, interstitial oedema and fibrosis) in the lungs. Tumour growth resulted in death of all animals with a median survival time of 32 days (Figure 1C). Growth of allogenic B16 melanoma was accompanied by a decreased and delayed alloreactive antibody production as compared to that induced by the injection of C57BL/6 PBMC together with B16 cells (Figure 1D).

### 3.2. Association of Low MHC Class I Expression with Allogenic B16 Melanoma Metastasis

**Figure 2:**
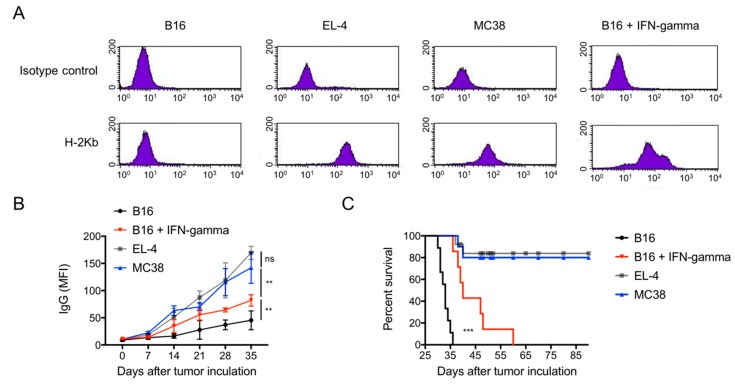
Major histocompatibility complex (MHC) class I expression and tumour metastasis in allogenic Balb/c mice. (**A**) Expression of MHC-I (H-2K^b^) on cultured B16, EL-4 and MC38 cells. Up-regulation of H-2K^b^ on B16 cells was seen after incubation with IFN-γ. (**B**) Serum anti-donor IgG in the mice after tumour cell i.v. injection. EL-4 and MC38 cells express high levels of MHC-I correlated with strong response of alloreactive antibody IgG (*p* < 0.001 vs. B16). Up-regulated expression of MHC-I on cultured B16 cells by IFN-γ resulted in an increased level of alloreactive anti-tumour IgG (*p* < 0.01 vs. non-stimulated B16). (**C**) Animal survival after *i.v.* injection of the indicated tumour cells. Mice received IFN-γ -stimulated B16 cells showed prolonged survival time as compared to that received non-stimulated B16 cells (*p* < 0.001). *n* = 10 mice in each group.

To test whether low MHC-I expression contributes to the metastatic growth of B16 melanoma in the lungs of allogenic Balb/c mice, we first evaluated tumour cell lines constitutively expressing high levels of MHC-I molecules (H-2K^b^) including EL-4 and MC38 tumour cells (Figure 2A). Following i.v. injection, both cancer cell types provoked a rapid alloreactive immune response characterized by production of high levels of alloreactive antibodies (Figure 2B) and rejection of tumour cells resulting in long-term tumour-free survival in >80% of tumour-challenged mice (Figure 2C). Secondly, injection of IFN-γ-stimulated B16 cells resulting in increased expression of MHC class I molecules (Figure 2A) was associated with an increased antibody response (Figure 2B) and significant improved survival of these mice (*p* < 0.05; Figure 2C). These data clearly suggest that there is a positive correlation between the low expression of MHC-I on B16 melanoma and disseminated tumour growth with inadequate alloreactivity.

### 3.3. Evaluation of Active and Passive Immunity in the Treatment of Allogenic B16 Melanoma Metastasis

**Figure 3:**
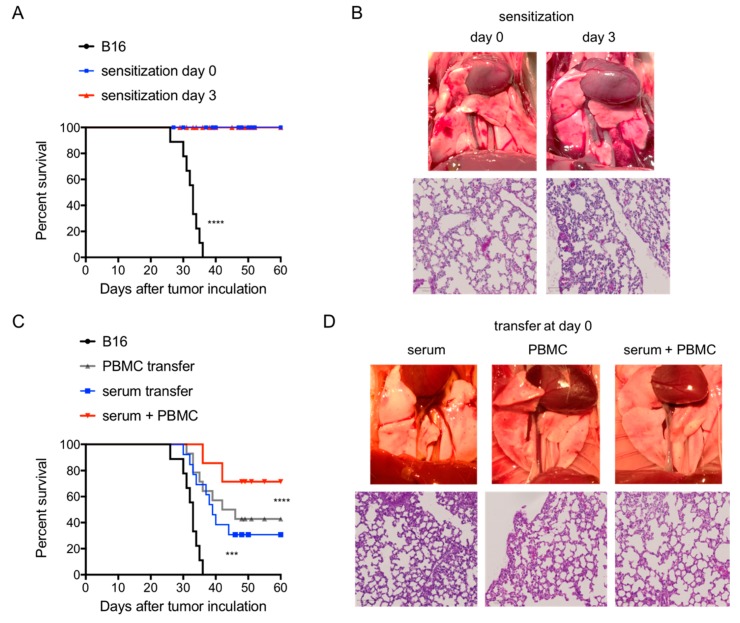

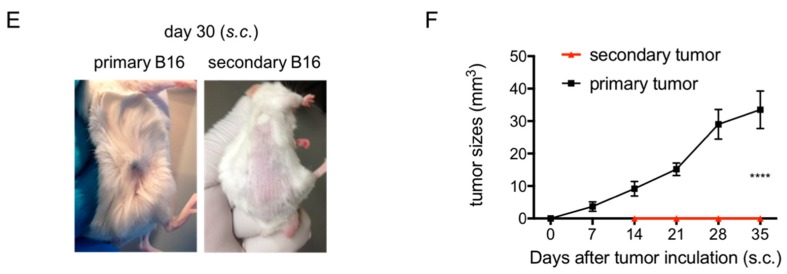
Treatment of metastasis by active and passive immunization. (**A**), Survival of Balb/c mice following *i.v.* injection of B16 cells with or without the indicated sensitization. (**B**) Clinical and histological image of B16 melanoma foci in the lungs of Balb/c mice (day 60 post B16 i.v. injection) with sensitization at time as indicated. Histology performed at day 60 following B16 i.v. injection showed that essentially no tumour foci was seen in surviving mice sensitized on day 0 and 3, except inflammation responses including immune cell infiltration, alveolar wall thickness, interstitial oedema and fibrosis. (**C**) Survival of Balb/c mice following *i.v.* injection of B16 cells with or without the indicated adoptive transfers. Alloreactive cytotoxic T lymphocytes (CTLs) or serum transfer prolonged animal survival as compared to that without transfer (*p* < 0.001). A combination of both resulted in a synergism as compared to individual transfer (*p* < 0.001). (**D**) Clinical and H&E staining image of B16 melanoma foci in the lungs of Balb/c mice receiving transfer as indicated. Histology was performed at day 60 following B16 i.v. injection showed that essentially no tumour foci was seen in surviving mice receiving activated CTLs or serum or a combination of both, despite singes of inflammation including immune cell infiltration, alveolar wall thickness, interstitial oedema and fibrosis. (**E**) Clinical images of melanoma growth day 30 post s.c. inoculation. Balb/c mice received B16 s.c. in naïve mice (left) versus mice carrying primary metastatic melanoma inoculated i.v. 14 days earlier (right). (**F**) Tumour volume after inoculation s.c. in naïve Balb/c mice versus mice carrying primary metastatic melanoma inoculated i.v. 14 days earlier.

We hypothesized that a delayed/decreased immune response induced by allogenic B16 cells is responsible for B16 melanoma metastasis. To evaluate this, Balb/c mice were immunized with normal PBMCs from C57BL/6 mice at day 0 or day 3 following i.v. B16 cell injection. This immunization resulted in an effective protection of animals from tumour-mediated killing (Figure 3A). Autopsy and histopathologic evaluation of long-term surviving mice (at day 60) showed no detectable tumour foci and presence of inflammation as evidenced by immune cell infiltration, alveolar wall thickness, interstitial oedema and fibrosis (Figure 3B). 

To further identify the cellular and humoral factors contributing to the anti-tumour effect, adoptive transfer of immunized serum, MLR-generated CTLs or a combination of both was performed at day 0, 2 and 4 post-i.v. tumour injection. Serum and CTLs transfers both prolonged survival to a median survival time of 40 and 44 days, respectively, as compared to survival in the control groups (MST = 32 days; *p* < 0.001) with 40–50% of animals surviving long-term (Figure 3C). Combination of CTLs and serum injection resulted in the long-term survival in 70% of animals (Figure 3C). In long-term surviving mice, tumour foci in the lungs (at day 60) were not detectable but signs of inflammation were present including immune cell infiltration, alveolar wall thickness, interstitial oedema and fibrosis (Figure 3D). 

To evaluate the function of the delayed/decreased allogenic immune response induced by primary metastatic melanoma, we performed secondary s.c. B16 cell inoculation 14 days after the primary i.v. melanoma inoculation. Normally, s.c. injected B16 melanoma cells undergo progression and regression in naïve allogenic mice (Figure 3E,F). S.c. injection of B16 melanoma cells after primary i.v. melanoma inoculation resulted in a complete elimination of s.c. injected secondary melanoma cells, while the primary melanoma metastasis was not interrupted (Figure 3E,F), indicating the development of a resistant TME in the primary melanoma foci.

### 3.4. Evaluation and Manipulation of the Tumour Microenvironment

**Figure 4:**
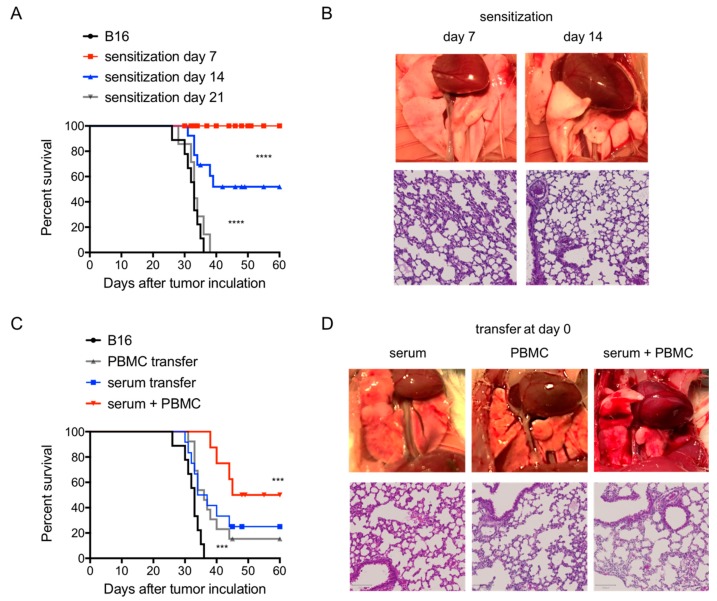
Treatment of tumour microenvironment (TME) by active and passive immunization. (**A**), Survival of Balb/c mice following *i.v.* injection of B16 cells with or without sensitization at different time as indicated. (**B**) Clinical and histological image of B16 melanoma foci (day 60 post B16 i.v. injection) in the lungs of Balb/c mice received sensitization at time as indicated. Essentially no tumour foci were detected in protected animals when sensitizations were performed at day 7 and day 14. Histology, performed at day 60 following tumour inoculation, showed that essentially no tumour foci was seen in protected mice receiving sensitizations, except inflammation responses including immune cell infiltration, alveolar wall thickness, interstitial oedema and fibrosis. (**C**) Survival of Balb/c mice following *i.v.* injection of B16 cells with or without the indicated transfer. Transfer of either activated cytotoxic T lymphocytes (CTLs) or serum at day 7 prolonged animal survival (*p* < 0.001). A combination of both showed synergy (*p* < 0.001. (**D**) Clinical and histological image of B16 melanoma foci in the lungs of Balb/c mice receiving adoptive transfer as indicated. Histology was performed at day 60 following tumour inoculation showing that essentially no tumour foci was seen in surviving mice receiving activated CTLs or serum transfer or a combination transfer of both, despite inflammation including immune cell infiltration, alveolar wall thickness, interstitial oedema and fibrosis. *n* = 10 mice in each group.

After establishing the presence of an immunosuppressive TME in metastatic melanoma, we first evaluated whether a boosted adaptive immune response by means of active and passive immunization would be able to overcome the TME. Active immunization was performed by injecting 5 × 10^6^ C57BL/6 spleen derived-PBMC in Balb/c mice. This active immunization at day 7 or 14 after i.v. melanoma inoculation protected animals from tumour-induced killing by 100% and 50%, respectively (Figure 4A). This protection was lost when the immunization was delayed to day 21 (Figure 4A). Surviving mice showed no tumour foci in the lungs at 60 days post-i.v. melanoma injection. In contrast, marked signs of an inflammatory response were noted consisting of immune cell infiltration, alveolar wall thickness, interstitial oedema and fibrosis (Figure 4B).

Next, we evaluated the efficacy of passive immunization to overcome the immunosuppressive TME through the adoptive transfer of activated CTLs and/or primed serum. Passive immunization was started on day 7 after i.v. melanoma injection when metastasis and TME were present and repeated every other day for a total of 3 doses. Transfer of serum or CTLs alone protected mice from tumour-mediated death with a prolongation of the median survival time from 32 days in control mice to 44 (*p* < 0.001) and 40 (*p* < 0.001) days, respectively (Figure 4C), with 20% animals surviving long-term in both passive immunization groups (Figure 4C). Passive immunization with both CTLs and serum resulted into a long-term survival of about 50% of mice (Figure 4C). Surviving animals showed no tumour foci at day 60 and signs of inflammation including immune cell infiltration, alveolar wall thickness, interstitial oedema and fibrosis (Figure 4D).

### 3.5. In Vitro Adaptive Immunity-Mediated Anti-Tumour Toxicity

**Figure 5:**
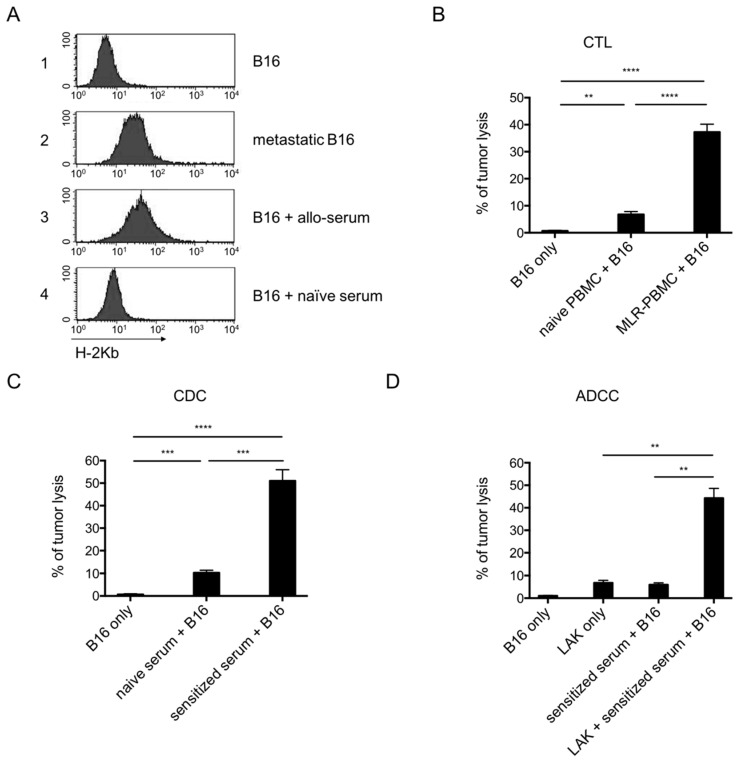
Cellular and humoral toxicity against tumour in vitro. (**A**) Levels of MHC-I (H-2K^b^) expression in the indicated tumour cells with or without the indicated treatment. Percentage of tumour lysis in CTL (**B**), complement-dependent cytotoxicity (CDC) (**C**) and antibody-dependent cellular cytotoxicity (ADCC) (**D**) assays. Data are representative of three separate experiments.

Despite the low MHC-I expression on cultured B16 cells, B16 melanoma showed a capacity to induce delayed/decreased allogenic immune responses and improved adaptive immunity resulted in improved tumour control, suggesting that MHC-I expression could be increased by proinflammatory signals in vivo. To elucidate this hypothesis, single cell suspensions were prepared from pulmonary metastasis. Indeed, the isolated B16 cells showed an upregulated expression of H-2K^b^ (Figure 5(A2)), as compared to cultured cells (Figure 5(A1)). The role of pro-inflammatory signals was confirmed in vitro by incubation of naïve B16 cells with of primed alloreactive serum which also resulted in an upregulation of MHC-I expression (Figure 5(A3)), as compared to naive serum (Figure 5(A4)).

In a next set of experiments, we evaluated the MHC-restricted alloreactivity against B16 cells in vitro. Incubation with IFN-gamma resulted in an increased MHC-I expression on B16 cells. T cell-mediated cytotoxicity was evaluated in vitro by CTL-mediated B16 cell lysis. CTLs generated in in vitro bulk MLR showed an increased ability to lyse B16 tumour cells as compared to naive PBMCs (37.3 ± 5.9% vs. 6.7 ± 2.2%; *p* < 0.0001; Figure 5B). We used the CDC assay to evaluate humoral anti-tumour reactivity. Serum from Balb/c mice immunized with C57BL6 PBMC resulted in increased lysis of target B16 tumour cells in the presence of complement (51.0 ± 10%) as compared to that induced by naïve serum (10.3 ± 2.7%) (*p* < 0.001; Figure 5C). The presence of alloreactive IgG was also demonstrated in the ADCC assay in which serum from Balb/c mice immunized with C57BL6 PBMC induced lysis of 44.3 ± 8.8% target B16 cell in the presence of LAK cells as compared to 6.3 ± 2.2% lysis by serum alone (*p* < 0.001; Figure 5D).

### 3.6. Th1 Versus Th2 Immune Paradigm

**Figure 6:**
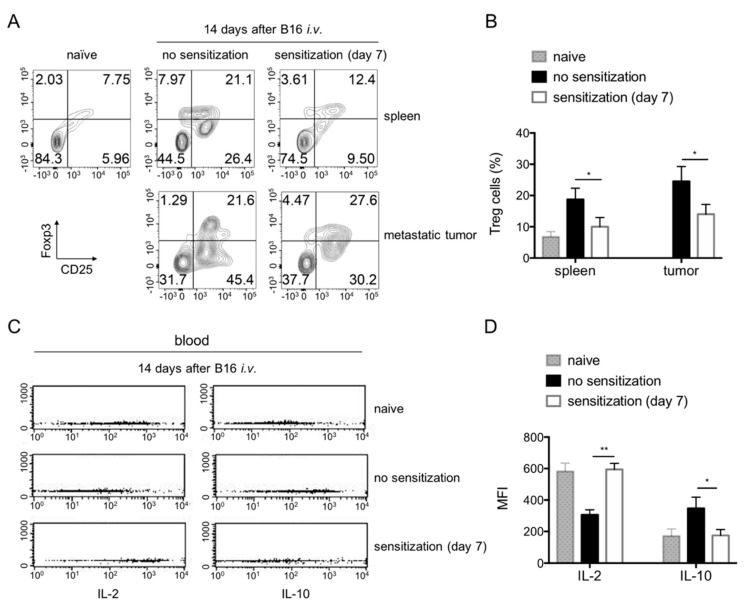
Alteration of TME associated immune paradigm after the active immunization. (**A**) Flow cytometry (FACS) profiles of Tregs (Foxp3^+^CD25^+^ cells gated on CD4^+^) in spleens and metastatic tumour in Balb/c mice 14 days after B16 i.v. injection with or without sensitization (at day 7). (**B**) Quantification of Tregs in Balb/c mice at conditions as described. In the non-sensitized mice, the spleen and tumour showed increased percentage of Tregs as compared to naïve mice (*p* < 0.05). Sensitization decreased the Tregs in both sites. (**C**) FACS profiles of IL-2 and IL-10 in the serum after stimulation of the mice with anti-CD3 antibody in vivo. (**D**) Quantification of the cytokine production showed that tumour bearing mice showed an increased IL-10 but decreased IL-2. Sensitization made these profiles converted. *n* = 4 mice in each group.

To study the development of TME, we analysed Tregs and the associated cytokines during tumour progression and immunotherapy. Fourteen days after i.v. tumour inoculation, disseminated tumour growth induced an increase of Tregs (Foxp3^+^CD25^+^ cells gated on CD4^+^) in the spleen from 6.8 ± 1.7% in naïve mice to 18.8 ± 3.6% in tumour-bearing mice (Figure 6A,B). The percentage of Tregs was up to 24.5 ± 4.8% in the tumour locations (Figure 6A,B). Concomitantly mice carrying metastatic tumours showed an increased serum IL-10/IL-2 ratio (Th2/Th1) after in vivo stimulation with anti-CD3 antibodies (Figure 6C,D). Immunization at day 7 with C57BL/6 PBMCs resulted in a decrease in Tregs to 10.0 ± 2.9% and 14 ± 3.2 in spleen and metastatic tumour, respectively (Figure 6A,B) and a decrease serum IL-10/IL-2 ratio (Figure 6C,D). Taken together, these data suggest that the TME and the Tregs-dominated immune response can be re-directed by an improved adaptive immune response directed against allogenic melanoma cells.

## 4. Discussion

Despite significant advances in anti-tumour immunotherapies, including checkpoint blockade regimens, durable responses occur only in a subset of cancer patients [1,2,3]. One of the major factors hindering current cancer immunotherapies is the TME that impedes the anti-cancer adaptive immune response [1,2,3]. Previous work from our group demonstrated that the TME extends beyond the tumour and can result in systemic immunosuppression [5]. Hence, there is an urgent need to better understand the features of TME and to develop new therapeutic strategies to overcome the TME [4,5,6]. Adaptive immune responses are central in the control of tumour growth and the development of the TME. Therefore, experimental models are needed to study adaptive anti-cancer immune responses. The model we used in this study is an allogeneic murine model where anti-cancer responses are driven by MHC-I directed adaptive immune responses and therefore this represents an ideal model to study the different components of the adaptive immune response involved in anti-cancer responses. 

Following i.v. B16 melanoma injection in allogeneic Balb/c mice, pulmonary metastases were observed from day 7 onwards resulting in 100% animal death. We demonstrated that tumour progression was driven by low expression of MHC-I resulting in insufficient anti-cancer adaptive immune responses [15,16,17,18,19,20], as evidenced by a delayed/decreased alloreactive antibody response. MHC-I expression acts as dominant tumour antigen in our model as evidenced by the following observations: (1) when MHC-I expression was up-regulated on B16 melanoma cells by pre-incubation with IFN-γ, the adaptive immune response was stronger and associated with alloreactive antibody production and the inhibition of tumour progression (Figure 2); (2) injection of EL-4 lymphoma (H-2K^b^) and MC38 colon cancer cells (H-2K^b^) which express normal levels of MHC-I antigens provoked strong alloreactive immune response and were rejected by the allogenic hosts (Figure 2). These results are in line with previous studies demonstrating that low MHC-I antigen expression is associated with tumour growth in allogenic mice [14,15,19,20]. Recent clinical trials in pre-treated small-cell lung cancer (SCLC) patients demonstrated that checkpoint blockade therapies resulted in response rates in only 10% and 25% of patients, which was lower compared to non-small-cell lung cancer (NSCLC) [10,11,12,21]. This was related to low MHC-I expression on tumour cells, resulting in insufficient antigen presentation to CTLs [21]. In addition, clinical benefits of checkpoint blockades have also been limited to a subgroup of melanoma patients [1,2,3] and this is most likely linked to a reduced immunogenicity of melanoma cells that prevents the induction of an effective adaptive immune response [7,8,9,10,22,23].

In an attempt to evaluate the extent to which adaptive immune response towards dominant tumour antigens is able to control disseminated pulmonary melanoma metastasis, we evaluated immunization with donor type PBMCs (expressing high levels of MHC-I antigens) at day 0 or day 3 after i.v. tumour inoculation on B16 melanoma growth. Immunization led to a restored alloreactive IgG response and prevention and/or elimination of metastases. Besides a humoral adaptive immune response, adoptive transfer experiments demonstrated the involvement of cellular adaptive immune response. Both donor-specific CTLs or serum from immunized animals could inhibit metastases in a time-sensitive manner. Co-transfer of both CTLs and primed serum resulted in synergistic effects, indicating the presence of multiple factors in combination leading to more potent anti-tumour effect in physiologic conditions in vivo [24,25,26,27]. In support of these results, previously data obtained in mouse models showed strong anti-melanoma cytotoxicity mediated by cellular and humoral immune response in vitro and in vivo [15,16,17].

Despite a failure to provoke a normal alloreactive immune response upon i.v. inoculation, metastatic melanoma cells were efficiently targeted by CTLs and antibodies following active or passive immunization. Up-regulation of MHC-I antigens is driven by inflammatory signals within the TME as evidenced by phenotype analysis. In vitro, similar findings were observed when B16 cells were incubated in the presence of primed alloreactive serum. Poor immunogenicity of B16 melanoma cells was associated with a delayed/decreased alloreactive immune response as evidenced by MHC-specific antibody formation. These responses were, however, able to completely prevent the outgrowth of secondary B16 melanoma cells inoculated s.c. The resistance of primary metastatic melanoma against anti-cancer adaptive immune responses suggest the development of an immunosuppressive TME in line with the previous findings [4,28].

To evaluate how the adaptive immune response interferes with the development of the TME, mice were immunized with allogenic PBMCs starting from day 7 after i.v. B16 melanoma injection (time point when metastasis and TME occurred). Immunization resulted in the eradication of established metastases and of the immunosuppressive TME in a time-sensitive manner. At an early stage of metastasis (e.g., immunization at day 7), metastases were completely eradicated leading to long-term tumour-free survival for all animals. Thereafter, the therapeutic effect progressively decreased (e.g., immunization at day 14) and completely disappeared when tumour foci became big and fusing to large masses (e.g., immunization at day 21). Similar effects can be achieved by passive immunization as well by adoptive transfer of alloreactive CTLs, primed serum or a combination of both. Both active and passive immunization therapeutics are clinically relevant [4,28,29,30]. These data suggest that immunization with the dominant tumour antigen can boost the anti-cancer adaptive immune responses and results in augmented tumour responses and improved outcomes.

Thus, the decreased immunogenicity of B16 melanoma cells contributes to decreased/delayed anti-cancer adaptive immune responses allowing for initial disseminated tumour growth in the lungs of allogenic hosts. By the time the adaptive immune response reaches a sufficient level to be protective against newly injected tumour cells, it is too late to control tumour progression due to the presence of an established immunosuppressive TME [4,28]. The failure of most therapies to combat established cancer seems to be related to a delayed occurrence of anti-tumour adaptive immune responses induced by the therapy in combination with tumour growth that rapidly outstrips the ability of the patient’s immune system to be effective to control the cancer [29,30]. However, the conversion of the Treg-dominated immune responses towards a Th1 type observed in the present study provided evidence that an established TME can be overcome by immunization and augmented anti-cancer adaptive immune responses. These findings are important to guide efforts to develop approaches to induce or enhance of host anti-cancer immune responses [4,5].

Both cellular and humoral immunity are involved in anti-tumour adaptive immune responses. The synergistic effect by co-transfer of CTLs and serum from immunized mice indicated the important interaction among immune cells and antibodies [30,31,32]. Our in vitro mechanistic studies elucidated multiple modes of action involved in tumour elimination, including CTL, CDC and ADCC. Previous reported studies showed that the anti-tumour effects of tumour binding antibodies and immune cells often act synergistic. For example, the rapid binding of injected tumour cells by these alloantibodies is a key element to activate dendritic cells (DC) and induce T cell-mediated anti-tumour immune responses, resulting in tumour eradication in experimental models of melanoma, pancreas, lungs and breast cancer [31,32,33].

## 5. Conclusions

Low MHC-I expression allows for disseminated pulmonary melanoma metastasis post i.v. inoculation and correlates with a delayed/decreased anti-cancer adaptive humoral and cellular immune responses. The formation of the TME, characterized by increased Th2/Th1 profiles, renders metastatic tumour resistant to the adaptive immune response. Improving this adaptive immune response by active and/or passive immunization is able to overcome the established metastatic tumour and to reverse Th2/Th1 profile in a time-sensitive manner. These findings suggest that the TME is not irreversible and adequate adaptive immunity is central in anti-tumour immunotherapy.
